# Risk Factors for Postictal Delirium in Geriatric Patients Undergoing Electroconvulsive Therapy: The Role of Lithium and Quetiapine

**DOI:** 10.31083/AP45431

**Published:** 2025-07-31

**Authors:** Shiling Wu, Kun Li, Jiang Long, Chenchen Zhang, Rui Li, Bochao Cheng, Minne Cao, Wei Deng

**Affiliations:** ^1^Department of Physiotherapy, Shandong Daizhuang Hospital, 272051 Jining, Shandong, China; ^2^Jining Key Laboratory of Neuromodulation, 272051 Jining, Shandong, China; ^3^Mental Health Center and Psychiatric Laboratory, The State Key Laboratory of Biotherapy, West China Hospital of Sichuan University, 610041 Chengdu, Sichuan, China; ^4^Department of Radiology, West China Second University Hospital of Sichuan University, 610041 Chengdu, Sichuan, China; ^5^Physical Integrated Diagnosis and Treatment Center, Affiliated Mental Health Center & Hangzhou Seventh People’s Hospital, Zhejiang University School of Medicine, 310058 Hangzhou, Zhejiang, China; ^6^Liangzhu Laboratory, MOE Frontier Science Center for Brain Science and Brain-machine Integration, State Key Laboratory of Brain-machine Intelligence, Zhejiang University, 311121 Hangzhou, Zhejiang, China

**Keywords:** geriatric patient, electroconvulsive therapy, postictal delirium, lithium, quetiapine

## Abstract

**Background:**

Postictal delirium (PID) is a significant and often underrecognized adverse effect associated with electroconvulsive therapy (ECT) in geriatric patients. Despite its clinical relevance, the specific risk factors contributing to the development of PID in this vulnerable population remain inadequately understood, which may affect treatment outcomes and patient safety.

**Methods:**

In this retrospective study, we analyzed data from 168 elderly patients who underwent ECT between 2009 and 2020 at a general hospital in China. Univariate analyses of sociodemographic and clinical characteristics were performed to identify variables for inclusion in a logistic regression model. Multiple binary logistic regression analysis was performed to determine the relationship between these variables and PID occurrence.

**Results:**

The incidence of PID was 20.8% (35/168) among the study cohort. Univariate analysis revealed statistically significant differences between PID and non-PID groups for lithium (*χ*^2^ = 6.67,* p* *=* 0.010), quetiapine (*χ*^2^ = 4.36, *p* = 0.037), number of ECT sessions (*U* = 3065.50, *p* = 0.003), and response rate (*χ*^2^ = 12.86, *p < *0.001). Logistic regression analysis demonstrated that lithium (odds ratio (OR) = 5.128; *p* = 0.009) and quetiapine (OR = 2.562; *p* = 0.024) were significantly associated with PID.

**Conclusion:**

Our findings indicate that lithium and quetiapine use significantly increase the risk of developing PID, underscoring the need for clinical vigilance. Careful consideration of these medications when planning ECT treatment is recommended to minimize the risk of postictal complications and optimize therapeutic outcomes.

## Main Points

(1) A total of 168 elderly patients who received electroconvulsive therapy were included in the study.

(2) The postictal delirium rate was 20.8% and the response rate was 72.6%.

(3) Lithium and quetiapine increased the risk of postictal delirium.

## 1. Introduction

Electroconvulsive therapy (ECT) remains one of the most effective treatments in psychiatry, showing significant efficacy in managing severe mental disorders like treatment-resistant depression and schizophrenia [1, 2, 3]. In recent years, its advantages in treating geriatric depression have become increasingly evident as attention to mental health in elderly patients has grown. Compared to pharmacotherapy, ECT not only achieves symptom remission more rapidly [4] but also has a higher remission rate [5], particularly among elderly patients. Moreover, ECT reduces the risk of suicide following hospital discharge, further emphasizing its importance in the treatment of geriatric mental disorders [6]. ECT has been established as a safe and effective alternative [7, 8].

Despite its proven efficacy, ECT is received by fewer than 1% of elderly patients with depression [9]. This may be due to concerns about the adverse effects of ECT [10]. Postictal delirium (PID) is one of the common side effects of ECT treatment [11, 12]. It is an acute state of confusion that typically occurs immediately after ECT, characterized by symptoms such as impaired consciousness, disorientation, and a decreased response [13]. While this condition typically resolves rapidly, it may persist for several hours or even days in some studies, posing potential risks to patients [14]. Among patients receiving ECT, 5.7% to 39.9% [15, 16, 17, 18] may experience PID, which can lead to adverse outcomes such as falls and the need for physical restraints due to irritability [19]. These complications can be potentially life-threatening, especially in elderly patients.

Several studies have examined risk factors for PID after ECT, identifying associations with various comorbidities including cerebrovascular disease [12], Parkinson’s disease [20], dementia [21], as well as specific medications such as etomidate [18] and lithium [11]. An interesting study found that complication rates after ECT was higher in older adults than in young adults (35% versus 18%), suggesting an age-dependent increase in risk, especially in patients with cardiovascular disease [21]. The increased risk in elderly patients may be due to multiple factors. As we age, the decline in cholinergic neurons weakens brain function, making individuals more prone to delirium [22]. Meanwhile, infections can trigger the release of inflammatory mediators and cytokines, which activate microglia and cause neuronal damage, potentially leading to acute delirium [23]. Additionally, older adults are more sensitive to hypoxia and metabolic disturbances, all of which increase the risk of delirium. Given that elderly patients exhibit increased susceptibility to delirium and consequently face elevated risks of adverse clinical outcomes [24], the delirium-inducing potential of ECT warrants heightened clinical attention and monitoring.

While previous studies have explored risk factors for PID after ECT in general populations, there is a notable paucity of research specifically focusing on elderly patients. The present study addresses this gap by investigating the risk factors for PID following ECT in elderly patients, utilizing a comprehensive database from a large hospital in China.

## 2. Materials and Methods

### 2.1 Study Design and Population

This was a single-center retrospective study based on real-world data. Inclusion criteria: Patients aged ≥60 years who received ECT at mental health center in a general hospital between January 2009 and December 2020; patients diagnosed by a psychiatrist with a mental disorder, such as depression or schizophrenia; patients have complete medical records and detailed ECT treatment records, including medication use, treatment parameters, post-treatment assessments, and more. Exclusion criteria: Patients who are also undergoing other physical therapies, such as transcranial magnetic stimulation. For subjects who received multiple ECT courses, only data from the first treatment course were included in the analysis.

### 2.2 ECT Treatment Protocol

All patients were treated with ECT by using the Thymatron System IV (SOMATICS, LLC, Lake Bluff, IL, USA). The initial seizure threshold was calculated using the half-age method [25], which is defined as the minimum electrical dose required to induce a seizure lasting at least 25 seconds on an Electroencephalogram. Bilateral temporal electrode placement was employed for all treatments. ECT was administered three times per week as the standard protocol. Subsequent treatment doses were set at 1.5 to 2.5 times the initial seizure threshold. The pulse width was 0.5 ms. Propofol served as the primary anesthetic agent, with etomidate and thiopental sodium available as alternatives under the supervision of the anesthesiologist and psychiatrist. All patients received penehyclidine hydrochloride for secretion management and succinylcholine for muscle relaxation.

### 2.3 Data Collection

Clinical data were extracted from electronic medical records, including demographic characteristics (age, gender, disease duration), anthropometric measurements (height, weight), anesthetic agents, ECT parameters (energy, current intensity, duration of stimulation, pulse width, numbers of treatment), diagnosis, etc.

Details of pharmacological treatment, including type and dose, were recorded one day before ECT. Specifically, we divided medications into the following categories: antidepressants (selective serotonin reuptake inhibitors [SSRIs], serotonin and norepinephrine reuptake inhibitors [SNRIs], tricyclic Antidepressants [TCAs], mirtazapine, agomelatine), mood stabilizers (lithium, valproate, lamotrigine), benzodiazepines, antipsychotics (olanzapine, clozapine, sulpiride, quetiapine, aripiprazole, risperidone, amisulpride, paliperidone).

In addition, neurological and endocrine comorbidities were documented due to their high prevalence of in geriatric populations. Physical comorbidities were divided into the following categories: hypertension, diabetes mellitus, hypothyroidism, Parkinson’s disease, dementia, history of cerebrovascular events (including both ischemic and hemorrhagic stroke).

We diagnosed PID using the delirium criteria from the Diagnostic and Statistical Manual of Mental Disorders, Fifth Edition, through a comprehensive review of course records and nursing records, including patient self-reports and direct observations from clinicians [26, 27]. These symptoms and management were documented in clinical and nursing records. Meanwhile, the clinical global impression-improvement (CGI-I) scale based on course record was used to evaluate the efficacy of ECT. The CGI-I scale ranges from 1 (very much improved) to 7 (very much worse). Scores of “1” (very much improved) and “2” (much improved) were considered indicative of treatment response. Both treatment response and PID assessments were conducted by two independent expert reviews (KL and JL). In cases of disagreement, the experts reviewed the clinical information jointly to reach a consensus.

### 2.4 Statistical Analysis

All variables were first subjected to univariate analysis. Chi-square test or Fisher’s exact test was conducted for categorical variables, and Mann-Whitney* U* test was performed for continuous variables, depending on whether the data conform to normal distribution or homogeneity of variance.

Multiple logistic regression analysis was performed on variables that were either identified as PID risk factors in previous studies or showed statistical significance ( *p* < 0.05) in univariate analysis. Prior to regression analysis, the linearity between the continuous independent variables and the logit of dependent variables was verified by employing the Box-Tidwell test, and multicollinearity among the independent variables was assessed using tolerance values or variance inflation factor. A regression model was constructed for the variables that met the requirements, and the forward likelihood ratio selection method was used for analysis. The Hosmer-Lemeshow test was used to evaluate the goodness of fit of the regression model, with *p* ≥ 0.05 indicating a good fit. The area under the curve (AUC) value was used to assess the model’s discriminative ability, with a larger AUC value indicating stronger discriminative ability. Statistical analyses were performed using SPSS (IBM SPSS, version 25, Chicago, IL, USA) and R software (Version 3.5.3, R Foundation for Statistical Computing, Vienna, Austria).

## 3. Results

### 3.1 Demographic and Clinical Characteristics

A total of 168 patients were enrolled in this study. Demographic and clinical characteristics are shown in Table 1. In our study, the median [interquartile range] age of the patients was 64.00 years [62.00, 68.00]. Among them, 110 patients (65.5%) were female. The median disease duration was 120.00 months [24.00, 240.00]. In terms of psychiatric diagnosis, 101 patients (60.1%) had major depressive disorder (MDD), 24 patients (14.3%) had bipolar disorder (BD), and 25 patients (14.9%) had schizophrenia. In this cohort, 35 patients (20.8%) developed PID after ECT. The overall response rate to ECT was 72.6%, with 122 patients showing clinical improvement.

**Table 1.** **Clinical and demographic characteristics**.

Characteristic		Value (n = 168)
Age (years)		64.00 [62.00, 68.00]
Disease duration (months)		120.00 [24.00, 240.00]
Body mass index		23.61 [21.87, 24.95]
Gender		
	Male	58 (34.5)
	Female	110 (65.5)
Diagnosis		
	Major depressive disorder	101 (60.1)
	Bipolar disorder	24 (14.3)
	Schizophrenia	25 (14.9)
	Others	18 (10.7)
Postictal Delirium		35 (20.8)
Response		122 (72.6)

Note: All variables are presented as median [interquartile range] or number (percentage).

### 3.2 Comparison Between Groups

Information on the clinical features of the PID and non-PID groups was shown in Table 2. There were no significant differences in age, disease duration, Body mass index and gender between PID and non-PID group. However, the non-PID group had a significantly higher response rate comparted to the PID group (χ^2^ = 12.86, *p* < 0.001).

**Table 2.** **Clinical features between postictal delirium and non-postictal delirium groups**.

Variable		PID group (n = 35)	Non-PID group (n = 133)	*U* or χ^2^	*p* value
Age^♢^		64.00 [63.00, 68.00]	64.00 [62.00, 68.00]	2288.50	0.878
Disease duration (months)^♢^		120.00 [24.00, 192.00]	120.00 [24.00, 252.00]	2179.50	0.563
Body mass index^♢^		23.73 [21.34, 25.53]	23.61 [22.02, 24.89]	1737.00	0.946
Gender^△^				0.19	0.665
	Male	11 (31.4)	47 (35.3)		
	Female	24 (68.6)	86 (64.7)		
Response^△^		17 (48.6)	105 (78.9)	12.86	<0.001
Anesthetics^△^				4.79	0.091
	Propofol	31 (88.6)	114 (85.7)		
	Etomidate	4 (11.4)	7 (5.3)		
	Thiopental Sodium	0 (0.0)	12 (9.0)		
Antidepressant drugs					
	SSRIs^△^	15 (42.9)	47 (35.3)	0.67	0.412
	SNRIs^△^	16 (45.7)	42 (31.6)	2.45	0.118
	TCAs^✩^	0 (0.0)	2 (1.5)	NA	1.000
	Mirtazapine^△^	2 (5.7)	5 (3.8)	0.27	0.607
	Agomelatine^✩^	0 (0.0)	1 (0.8)	NA	1.000
Mood stabilizers					
	Lithium^△^	6 (17.1)	6 (4.5)	6.67	0.010
	Valproate^△^	1 (2.9)	8 (6.0)	0.55	0.460
	Lamotrigine^✩^	1 (2.9)	0 (0.0)	NA	0.208
Benzodiazepines^△^		19 (54.3)	54 (40.6)	2.11	0.146
Antipsychotics					
	Olanzapine^△^	18 (51.4)	68 (51.1)	<0.01	0.975
	Clozapine^△^	3 (8.6)	16 (12.0)	0.33	0.565
	Sulpiride^△^	2 (5.7)	3 (2.3)	1.15	0.284
	Quetiapine^△^	14 (40.0)	30 (22.6)	4.36	0.037
	Aripiprazole^✩^	0 (0.0)	2 (1.5)	NA	1.000
	Risperidone^△^	1 (2.9)	13 (9.8)	1.74	0.188
	Amisulpride^△^	0 (0.0)	5 (3.8)	1.36	0.244
	Paliperidone^△^	0 (0.0)	5 (3.8)	1.36	0.244
Physical comorbidities					
	Hypertension^△^	13 (37.1)	30 (22.6)	3.10	0.079
	Diabetes Mellitus^△^	6 (17.1)	21 (15.8)	0.04	0.846
	Hypothyroidism^△^	1 (2.9)	6 (4.5)	0.19	0.663
	Parkinson disease^✩^	0 (0.0)	4 (3.0)	NA	0.581
	Dementia^✩^	0 (0.0)	4 (3.0)	NA	0.581
	History of CI or CH^△^	4 (11.4)	19 (14.3)	0.19	0.662
Electrical parameters					
	Energy (J)^♢^	50.00 [50.00, 55.00]	50.00 [50.00, 55.00]	2601.00	0.265
	Stimulus duration (s)^♢^	7.00 [7.00, 7.70]	7.00 [6.70, 7.70]	2898.00	0.891
	Current (mA)^♢^	900.00 [890.00, 900.00]	900.00 [890.00, 900.00]	2587.50	0.271
	Number of ECT^♢^	5.00 [3.00, 7.00]	6.00 [5.00, 8.00]	3065.50	0.003
Diagnosis^△^				8.58	0.035
	MDD^△^	22 (62.9)	79 (59.4)	0.14	0.710
	Bipolar disorder^△^	9 (25.7)	15 (11.3)	4.72	0.030
	Schizophrenia^△^	1 (2.9)	24 (18.0)	5.05	0.025
	Others^△^	3 (8.6)	15 (11.3)	0.21	0.645

Notes: All variables are presented as median [interquartile range] or number (percentage). ^△^ Chi-squared test; ^♢^Mann–Whitney *U* test; ^✩^ Fisher’s exact test. Abbreviations: PID, postictal delirium; SSRIs, selective serotonin reuptake inhibitors; SNRIs, serotonin and norepinephrine reuptake inhibitors; TCAs, tricyclic antidepressants; NA, Not Applicable; CI, cerebral infarction; CH, cerebral hemorrhage; ECT, electroconvulsive therapy; MDD, major depressive disorder.

There were no statistically significant differences in the types of anesthetics used, energy levels, stimulation duration and current intensity used in ECT (Table 2). The PID group, however, received fewer ECT compared to the non-PID group (*U* = 3065.50, *p* = 0.003).

Antidepressants and benzodiazepines did not differ statistically in medication use between the PID and non-PID groups. Among mood stabilizers, valproate and lamotrigine did not differ between groups. But interestingly, lithium usage was showed a significant difference (χ^2^ = 6.67, *p* = 0.010). In terms of antipsychotics (olanzapine, clozapine, sulpiride, aripiprazole, risperidone, amisulpride, and paliperidone), there was no statistically significant difference between the PID and non-PID groups. However, quetiapine was the only antipsychotics with statistically significant differences between PID and non-PID groups (χ^2^ = 4.36, *p* = 0.037).

Moreover, there were no significant differences in comorbidities of physical disease between groups. The present study found statistically significant differences in psychiatric diagnosis between groups by univariate analysis (χ^2^ = 8.58, *p* = 0.035). Specifically, the differences in bipolar disorder (χ^2^ = 4.72, *p* = 0.030) and schizophrenia (χ^2^ = 5.05, *p* = 0.025) between the two groups were statistically significant.

### 3.3 Multiple Logistic Regression Model

In our preliminary analysis, we observed that the number of ECT sessions did not show a linear relationship with the logit of PID incidence (*p* = 0.007). This lack of linearity persisted even after applying a logarithmic transformation to the independent variable, with no significant improvement. Additionally, considering that the PID group received fewer ECT sessions, this may be due to the occurrence of PID necessitating the premature termination of the ECT course. As a result, the number of ECT sessions was excluded from the final regression model.

We performed a multiple logistic regression analysis to identify risk factors for PID, using psychiatric diagnosis, lithium, and quetiapine as independent variables. The analysis revealed significant associations between PID and both lithium (odds ratio (OR) = 5.128; 95% confidence interval (CI) [1.492–17.622]; *p* = 0.009) and quetiapine (OR = 2.562; 95% CI [1.134–5.788]; *p* = 0.024). Patients receiving either lithium or quetiapine alongside ECT had a higher risk of developing PID. Lithium was primarily prescribed for mood disorders, including MDD and BD, while quetiapine was also mainly used for mood disorders, accounting for 76.7% of cases.

The Hosmer-Lemeshow test yielded a result indicating good model fit (*p* = 0.872). We also calculated the AUC values for each predictive indicator and the combined AUC value. The AUC value for lithium was 0.563, and for quetiapine, it was 0.587. When lithium and quetiapine were combined, however, the AUC value increased to 0.709.

## 4. Discussion

The purpose of this study was to investigate the risk factors for PID after ECT in a cohort of 168 elderly patients. Among the study population, 35 patients (20.8%) developed PID. Univariate analysis revealed that lithium, quetiapine, response rates, diagnosis and number of ECT sessions were statistically different between groups. There were no significant differences between groups in demographics, antidepressants, anesthetics, benzodiazepines, physical comorbidities, or electrical parameters. Multiple logistic regression analysis identified lithium and quetiapine as significant predictors of PID occurrence. The AUC values indicated that the predictive ability of lithium or quetiapine alone for PID was limited. However, the combined use of lithium and quetiapine significantly improved the predictive ability.

The majority of patients treated with ECT were diagnosed with mood disorders, including MDD and BD, accounting for 74.4% of cases. This finding aligns with a large retrospective study from China that reported affective disorders accounted for 81.5% of elderly people treated with ECT [28]. This may be attributed to ECT’s established efficacy and favorable response rates in geriatric affective disorders. The observed response rate of 72.6% in our study is consistent with previous findings [29]. Recent investigations, including a clinical study [30] and meta-analysis [31] focusing on bipolar depression, reported response rates of 80.2% and 77.1% respectively. Additionally, a study specifically examining older adults reported a high response rate of 80.8% to ECT [7]. These studies suggested that ECT is effective in the elderly, especially for affective disorders.

However, adverse effects warrant careful consideration, as evidence by the 20.8% incidence of PID in our cohort. Previous studies involving elderly patients have documented overall complication rates around 50% [7]. Our findings corroborate previous studies indicating that the incidence of complications, especially PID, increases with age — a phenomenon attributable to the heightened complexity of medical comorbidities in geriatric populations. While several previous studies and reviews have suggested associations between PID and factors such as anesthetics [18, 32], energy intensity and physical comorbidities [12], our study failed to demonstrate such correlations. This discrepancy may be attributed to the study’s limited focus on specific conditions such as Parkinson’s disease, and future investigation with larger sample sizes are warranted for validation.

In accordance with our expectations, this study revealed that lithium increased the risk of PID after ECT, a finding that aligns with mainstream research findings. Several studies [11, 33] and case reports [34] have documented lithium’s role in elevating PID risk after ECT. However, some studies have reported contradictory findings, suggesting no significant association between lithium and PID risk [35, 36]. These discrepancies may be attributed to substantial heterogeneity among study populations and variations in sample sizes. Notably, a recent large-scale retrospective study of 64,728 hospitalized patients examining the adverse effects of concurrent lithium and ECT administration revealed that this combination was associated with an 11.7-fold increase in PID incidence compared to ECT monotherapy [11]. The mechanism underlying this interaction may be related to lithium’s intracellular-mediated toxic effects [37]. During ECT-induced epileptiform activity, enhanced sodium channel activation facilitates the intracellular transport of extracellular lithium [38], potentially amplifying its toxic effects. Consequently, serum lithium levels may paradoxically decrease rather than increase after ECT. These findings underscore the importance of monitoring serum lithium concentration prior to ECT administration. In cases where ECT is clinically indicated, clinicians should consider reducing lithium dosage to maintain serum concentrations below the recommended threshold of 0.7 mmol/L [39].

A noteworthy finding of our investigation was the association between quetiapine administration and increased PID risk, corroborating results from a recent retrospective study [40]. This association may be attributed to quetiapine’s binding affinity for adrenergic receptors, which can precipitate orthostatic hypotension, particularly in geriatric populations [41, 42]. The predominant use of both lithium and quetiapine in affective disorders may explain the elevated PID incidence observed among patients with these conditions in our cohort.

The AUC values for lithium and quetiapine were 0.563 and 0.587, showing that each drug alone has limited predictive power for PID. But when combined, the AUC rose to 0.709, showing much better predictive power. This could be because the two drugs interact. Together, they might have a bigger effect on PID risk than either one alone. They might work together to affect brain chemicals like dopamine and serotonin, and boost anticholinergic effects. This could lead to big changes in neurotransmitter levels and central cholinergic function, raising the risk of delirium [43, 44]. Also, when used alone, the sample size was uneven (35 cases in the PID group and 133 cases in the non - PID group). This imbalance might have made the model favor the non - PID group, lowering the AUC. But the multifactorial model, by including multiple variables, can better capture PID risk factors. It can partly offset the impact of sample size imbalance, improving predictive power. These findings suggest that when using lithium and quetiapine in elderly patients, especially those receiving ECT, the risks of combined use should be carefully evaluated.

This study is the first to investigate risk factors for PID in elderly patients receiving ECT. However, the study has some limitations. Above all, the retrospective study design introduces inherent constraints. Despite employing univariate and regression analysis, there were some unmeasured residual confounding variables, such as medication interactions, uncommon ECT dose adjustment protocols, and cognitive impairment in elderly patients. A prospective study design is required for further exploration. Second, while our findings implicate lithium and quetiapine in increased PID risk, these medications were predominantly prescribed for MDD and BD in our cohort, potentially limiting their predictive value for other psychiatric conditions. Further disease-specific analyses are warranted to address this limitation. In addition, our study did not incorporate PID severity assessments, such as Memorial Delirium Assessment Scale [45], which could provide more nuanced insights into ECT-related adverse effects. Fourthly, the absence of data regarding ECT electrode placement sites and seizure duration parameters limits our ability to evaluate these potentially significant variables, particularly given previous research linking electrode placement to PID occurrence [46]. Lastly, the single-center design and relatively modest sample size underscore the need for validation through multi-center studies with larger patient populations.

## 5. Conclusion

This study identified lithium and quetiapine as significant risk factors for PID after ECT. These findings have important clinical implications, emphasizing the need for careful monitoring of patients receiving these medications who require ECT treatment. Given the elevated PID risk associated with these agents, clinicians should exercise heightened vigilance and consider implementing rigorous drug concentration monitoring protocols prior to ECT administration.

## Data Availability

The data for this submission in this study is not be publicly available due to privacy.
